# Supplementary material for: CAGEE: Computational Analysis of Gene Expression Evolution
Source: Mol Biol Evol. 2023 May 9;40(5):msad106. doi: 10.1093/molbev/msad106 (PMC10195155; doi:10.1093/molbev/msad106)
Supplement: msad106_Supplementary_Data [file msad106_supplementary_data.zip › FIgures_revised_supp.pdf]

Supplementary Figure 1

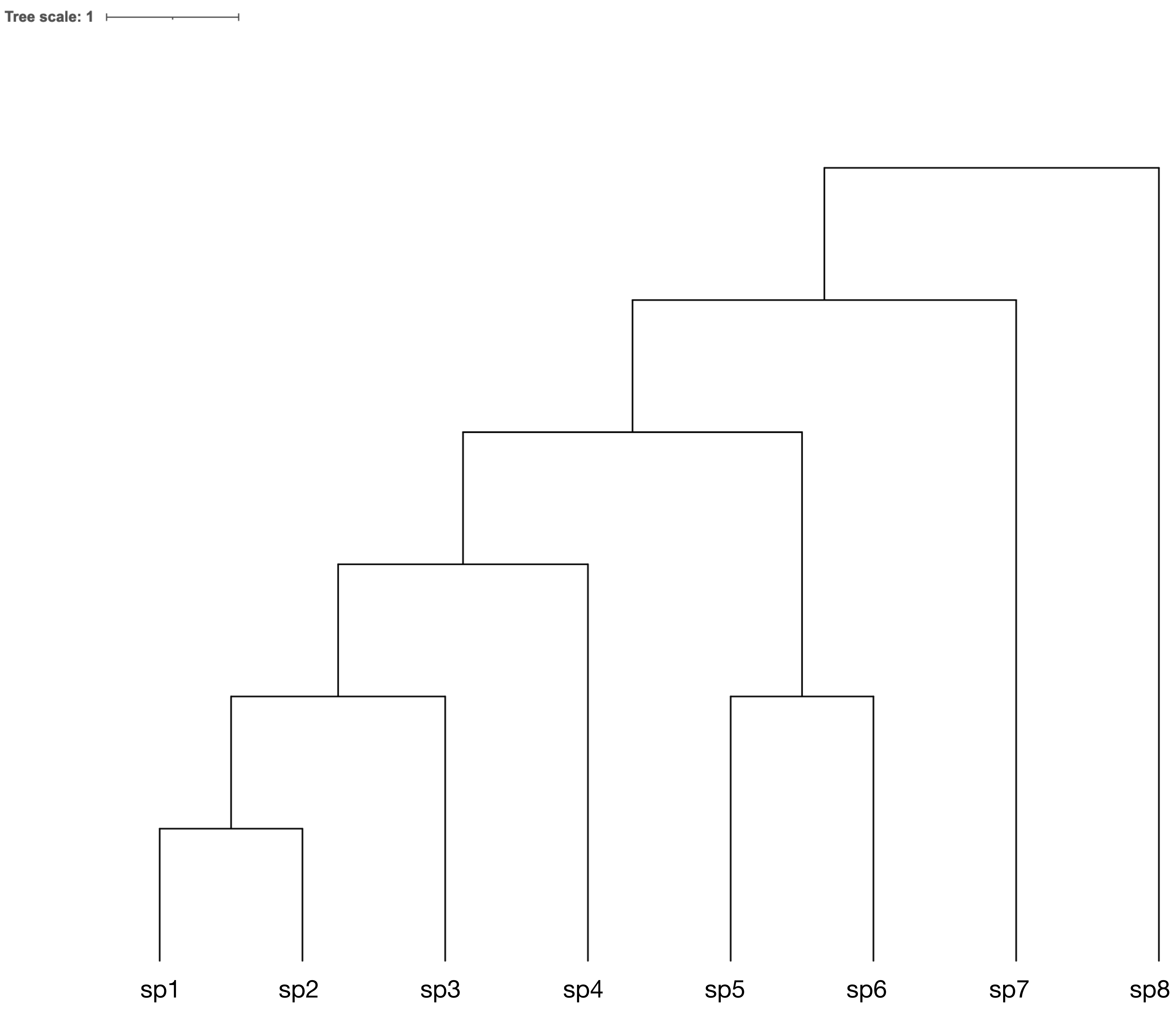

Supplementary Figure 2A

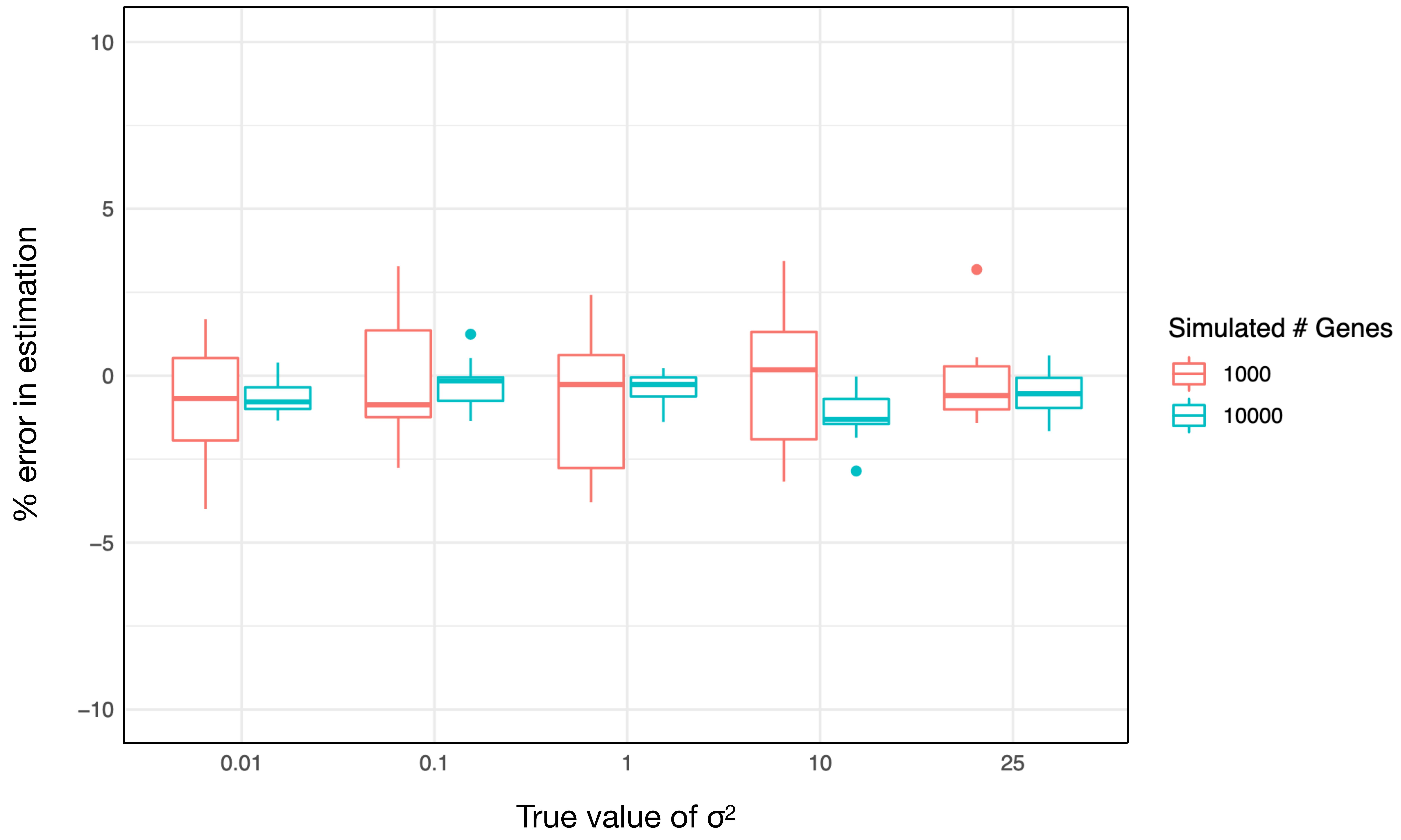

Supplementary Figure 2B

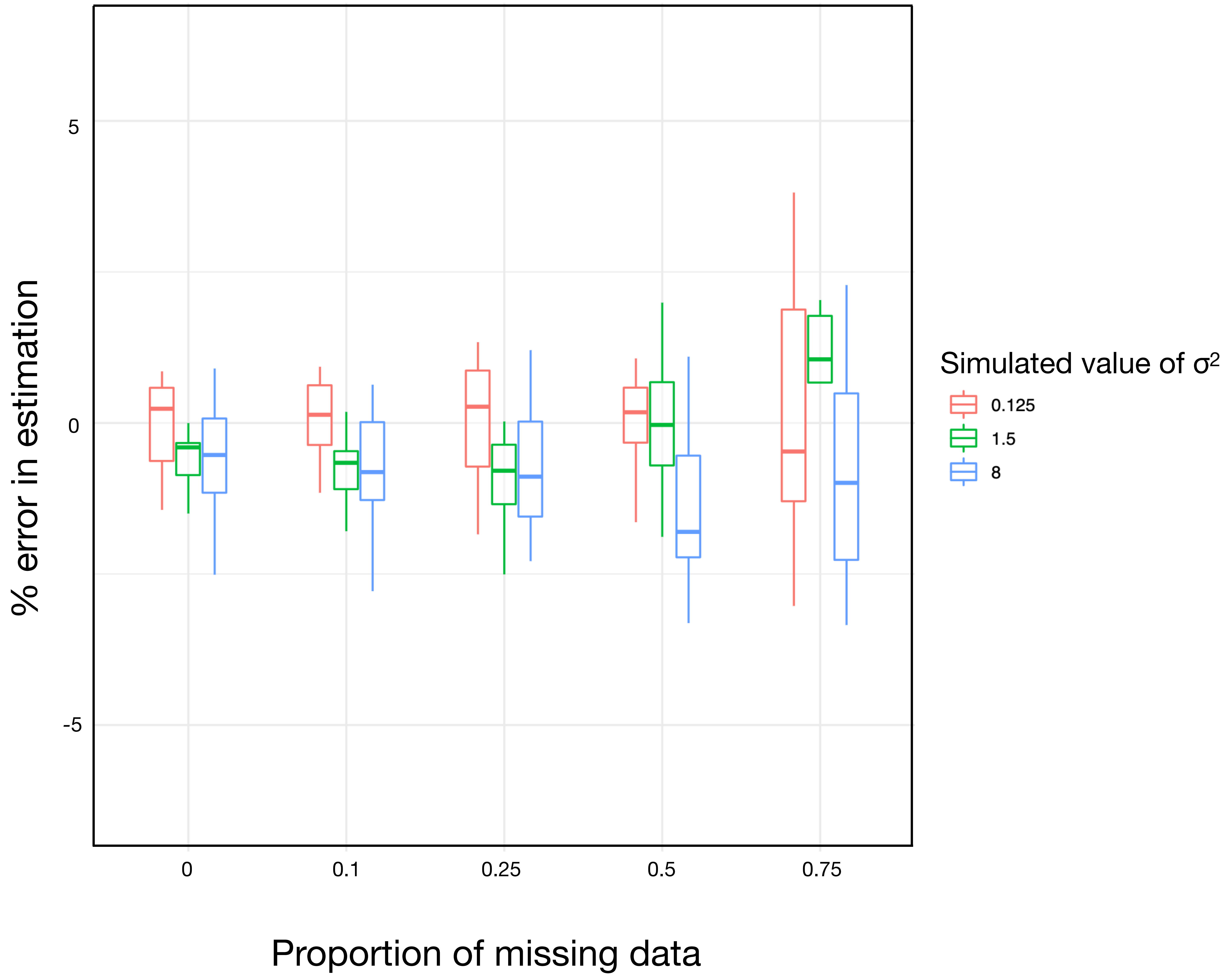

Supplementary Figure 2C

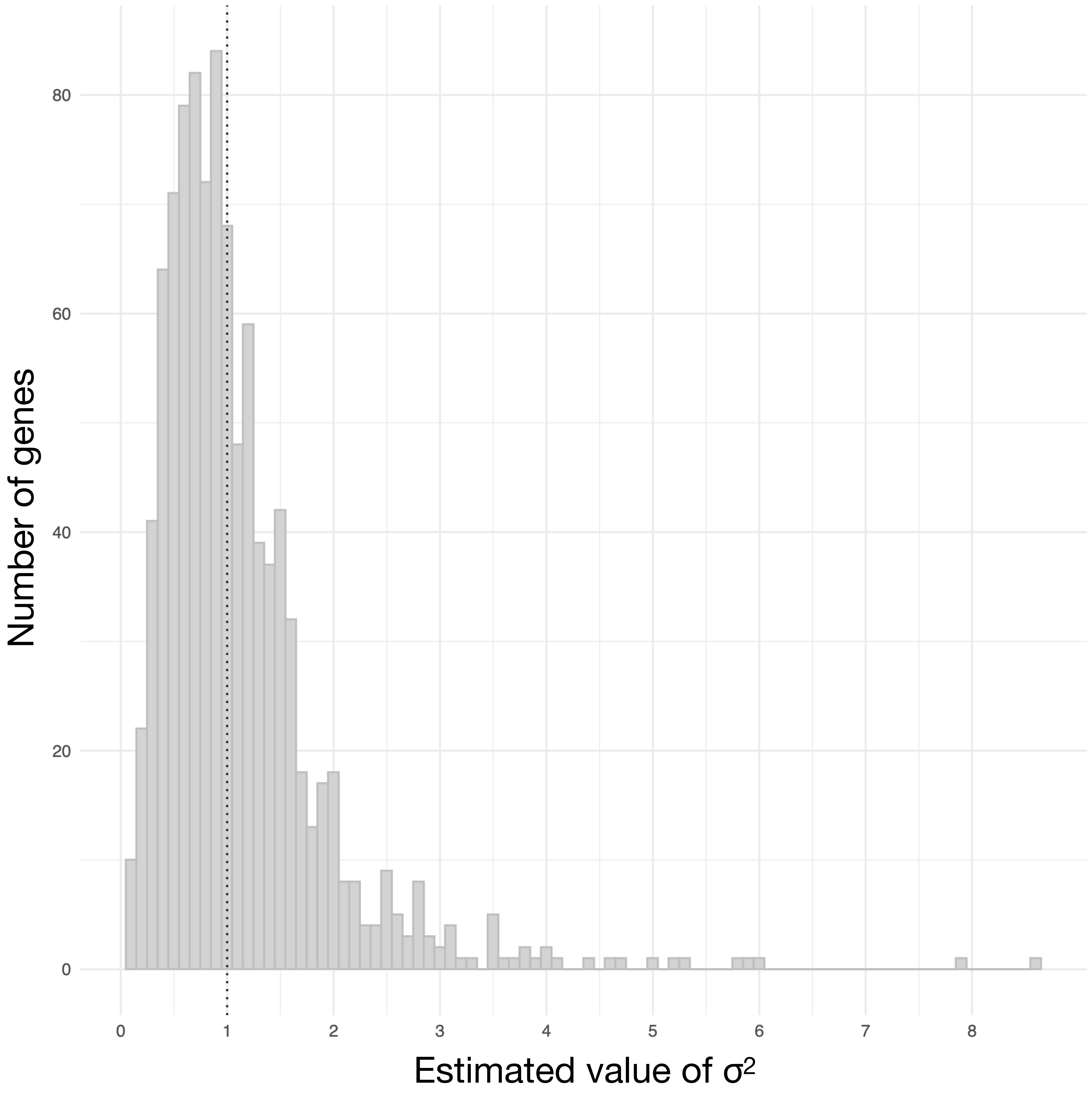

Supplementary Figure 2D

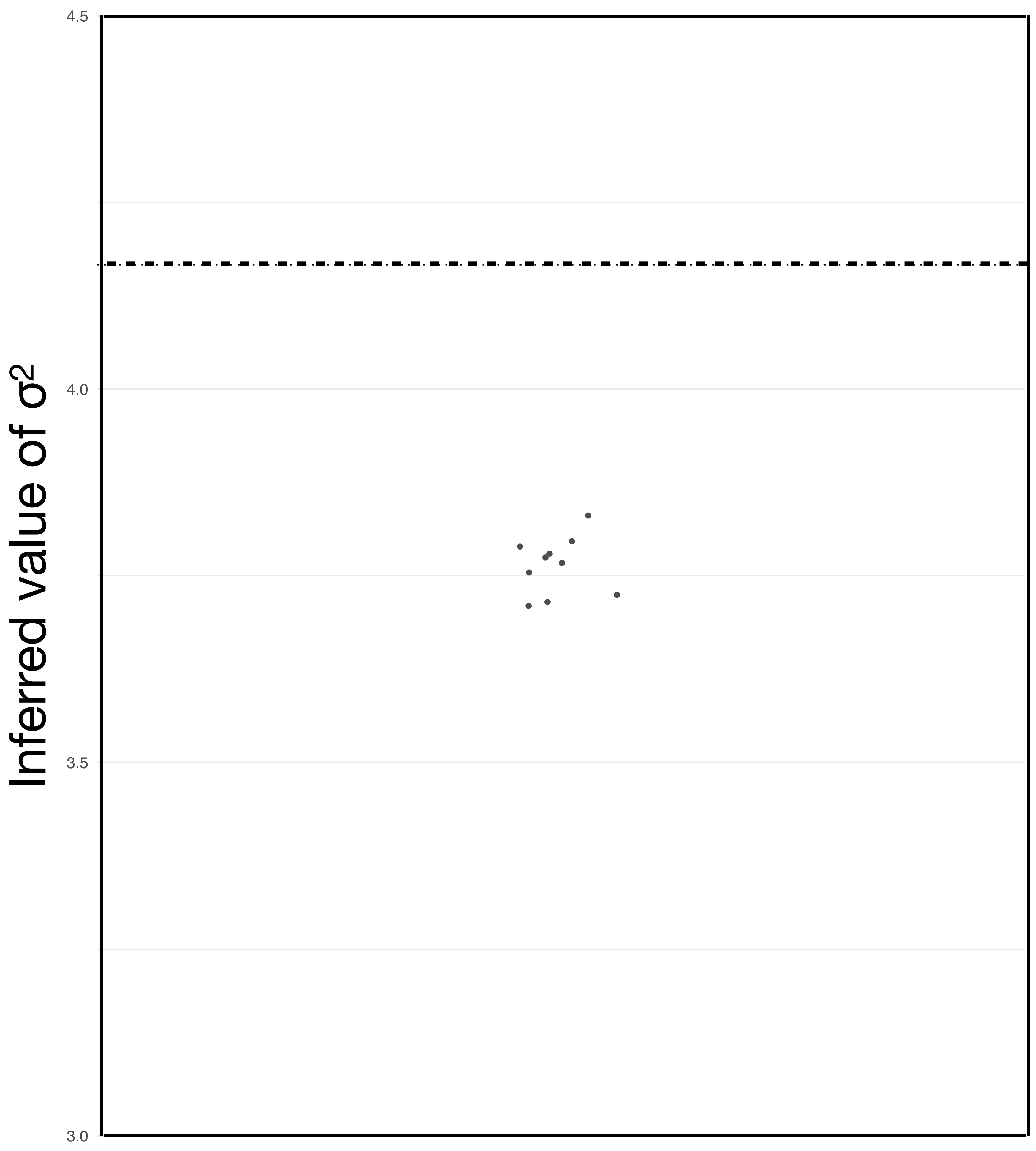

Supplementary Figure 3

A)

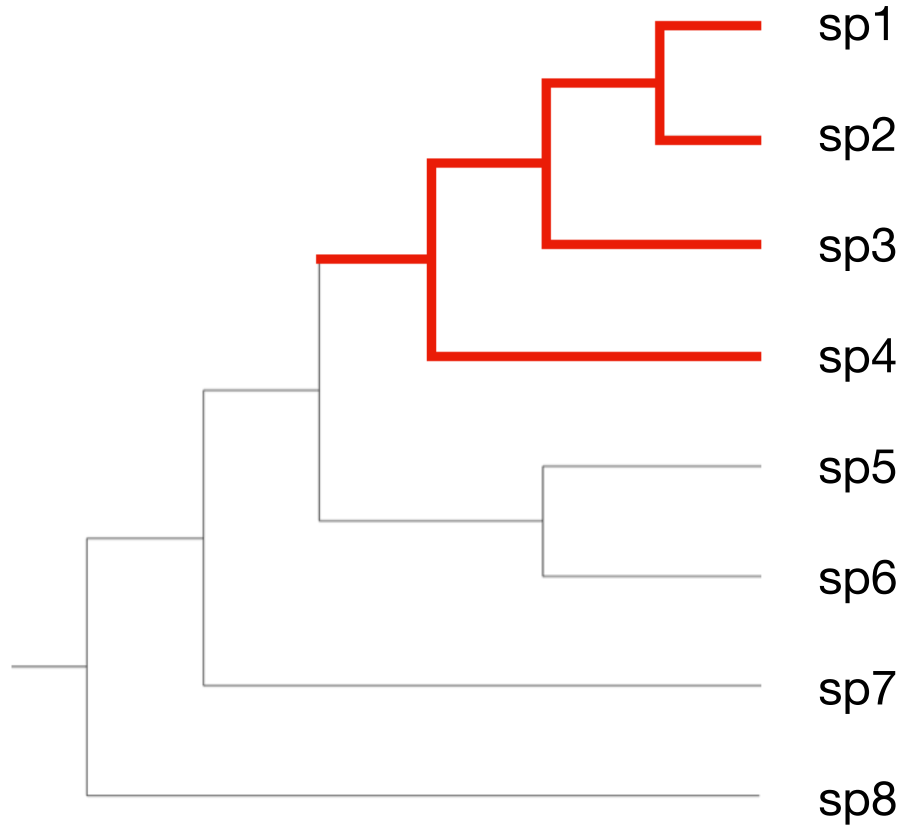

B)

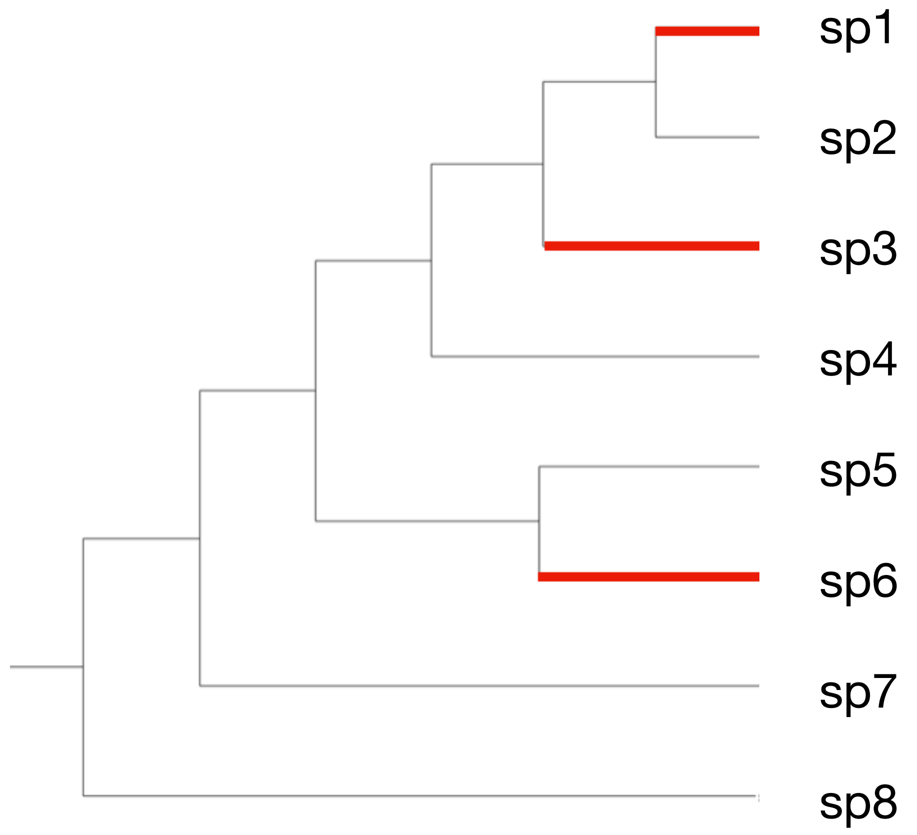

Supplementary Figure 4

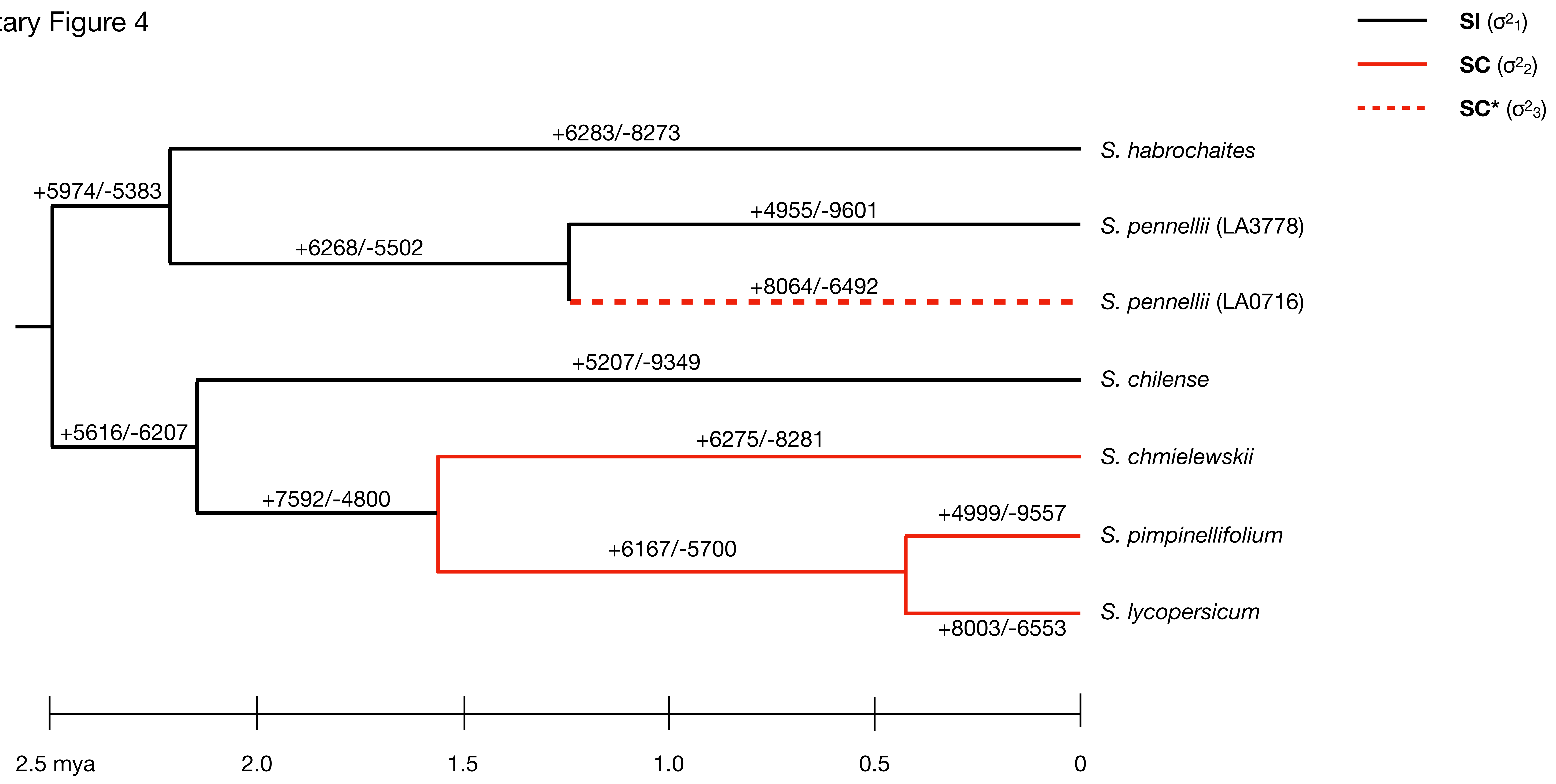

Supplementary Figure 5

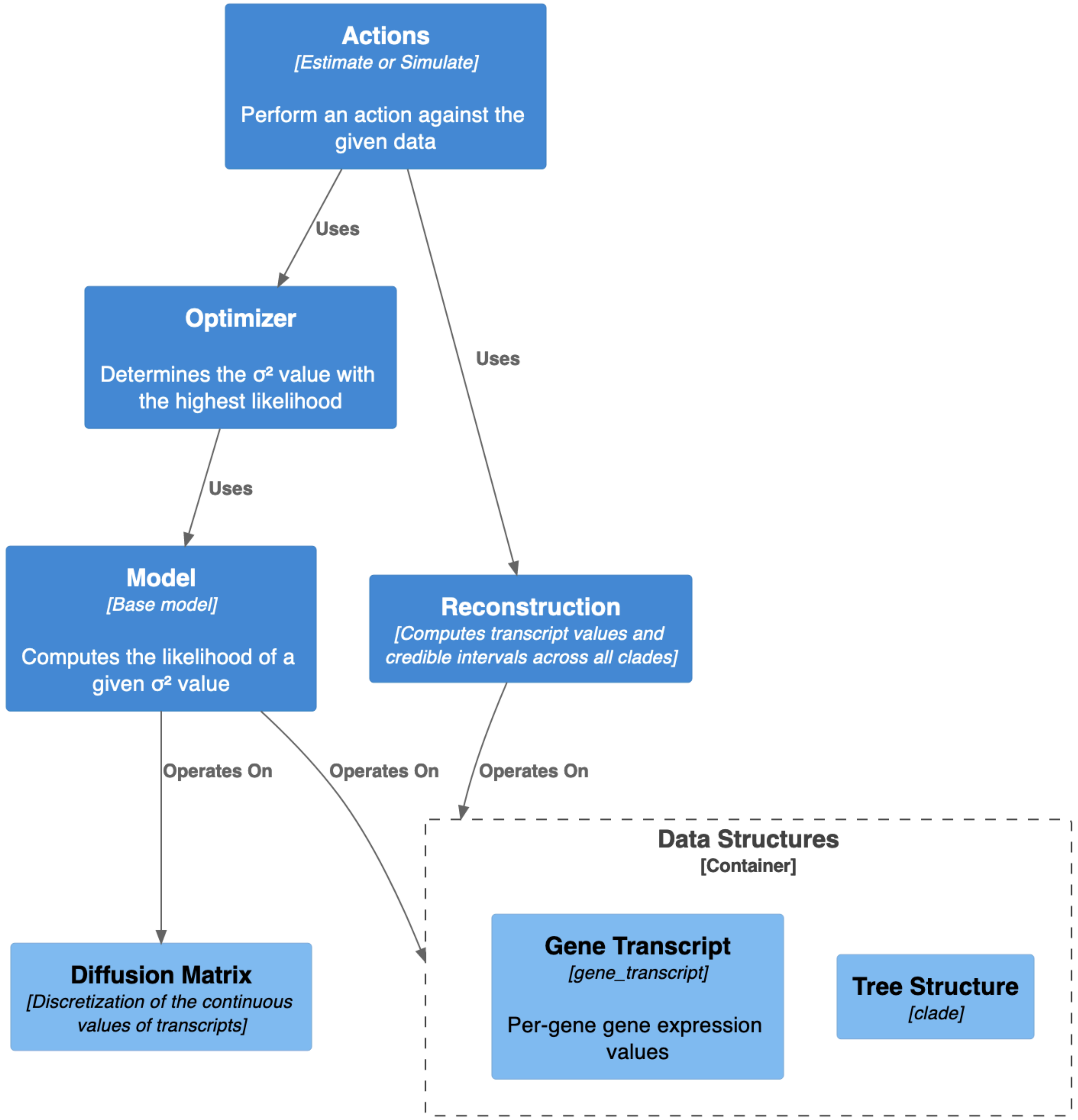

Supplementary Table 1

Set of σ^2 values	Replicate	Simulated σ^2	Inferred σ^2	% error
0.2, 1.0	1	0.2	0.1926803	-3.66%
		1.0	1.0024905	0.249%
	2	0.2	0.1943218	-2.84%
		1.0	1.0054018	0.54%
	3	0.2	0.2031335	1.57%
		1.0	0.9577028	-4.23%
	4	0.2	0.1967767	-1.61%
		1.0	0.9996745	-0.0325%
	5	0.2	0.1924732	-3.76%
		1.0	1.0075440	0.754%
	6	0.2	0.2004951	0.248%
		1.0	1.0242412	2.42%
	7	0.2	0.1944284	-2.79%
		1.0	0.9733314	-2.67%
	8	0.2	0.2024781	1.24%
		1.0	0.9884935	-1.15%
	9	0.2	0.2018622	0.931%
		1.0	1.0382544	3.83%
	10	0.2	0.1956765	-2.16%
		1.0	1.0065474	0.655%
1.0, 3.0	1	1.0	0.9451556	-5.48%
		3.0	3.0287977	0.96%
	2	1.0	0.9769290	-2.31%
		3.0	3.1000229	3.33%
	3	1.0	0.9861053	-1.39%
		3.0	2.9452423	-1.83%
	4	1.0	0.9724728	-2.75%
		3.0	2.9203197	-2.66%
	5	1.0	0.9226307	-7.74%
		3.0	2.9930805	-0.231%
	6	1.0	0.9939074	-0.609%
		3.0	2.8856497	-3.81%
	7	1.0	0.9871113	-1.29%
		3.0	2.9705234	-0.983%
	8	1.0	0.9684494	-3.16%
		3.0	2.9187219	-2.71%
	9	1.0	0.9667607	-3.32%
		3.0	3.1521085	5.07%
	10	1.0	1.0059515	0.595%
		3.0	3.0232185	0.774%
5.0, 10.0	1	5.0	4.7464719	-5.07%
		10.0	9.9814701	-0.185%
	2	5.0	4.8651485	-2.7%
		10.0	10.0907926	0.908%
	3	5.0	4.7598429	-4.8%
		10.0	9.8058231	-1.94%
	4	5.0	4.9265027	-1.47%
		10.0	10.0593675	0.594%
	5	5.0	4.9239001	-1.52%
		10.0	9.9818898	-0.181%
	6	5.0	4.8684289	-2.63%
		10.0	9.8020147	-1.98%
	7	5.0	4.8752468	-2.5%
		10.0	10.0378953	0.379%
	8	5.0	4.9042695	-1.91%
		10.0	9.8541603	-1.46%
	9	5.0	4.6019327	-7.96%
		10.0	9.9286600	-0.713%
	10	5.0	4.8589572	-2.82%
		10.0	9.8313460	-1.69%
